# Supplementary material for: Single-cell lineage analysis reveals genetic and epigenetic interplay in glioblastoma drug resistance
Source: Genome Biol. 2020 Jul 15;21:174. doi: 10.1186/s13059-020-02085-1 (PMC7364565; doi:10.1186/s13059-020-02085-1)
Supplement: Supplementary file 1 — Additional file 1. Supplemental Figures and Legends (Figs. S1-S6). Contains compiled supplementary figures and legends referenced in the main text. [file 13059_2020_2085_MOESM1_ESM.pdf]

SUPPLEMENTARY FIGURES

Supplementary Fig. S1

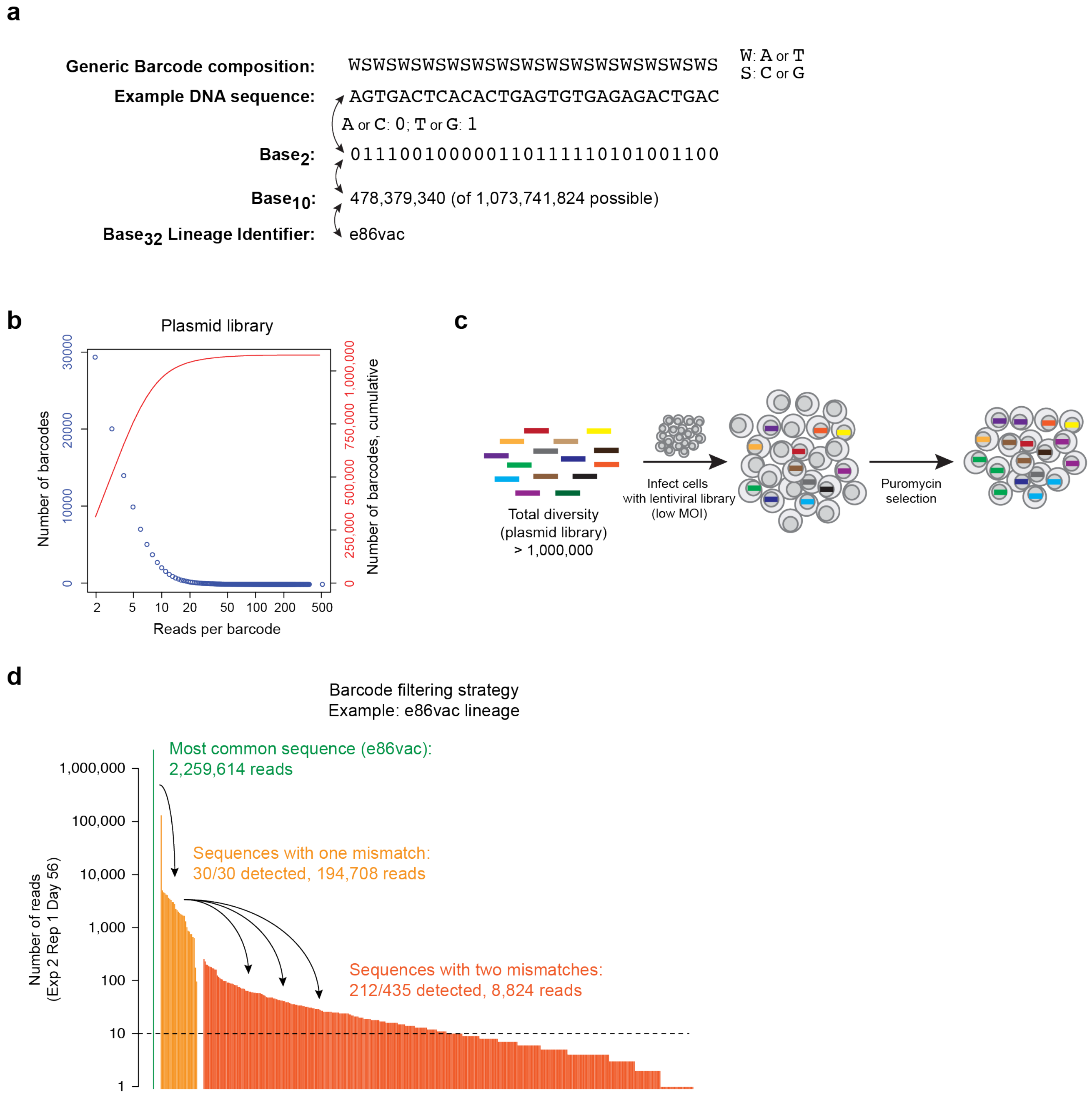

Figure S1.

a) Schematic depicts an example of lineage ID conversion. The 30bp DNA lineage barcode is first converted to a binary (base 2) representation. This binary representation is further converted to base 10 or base 32, which for simplicity are used throughout the manuscript. The base 32 converted barcodes can be readily converted back to the original DNA sequence.

b) Plot depicts barcode distributions in plasmid library. Blue circles indicate the number of barcodes (left axis) that were detected with a given number of reads (x axis). The red line indicates the cumulative number of barcodes (right axis) that were detected with a given number of reads. More than one million unique barcodes were detected in the plasmid library with two or more reads.

c) Schematic depicts the introduction of plasmid barcode library to cell population and selection for barcode-bearing cell population.

d) Barcode filtering strategy, exemplified for the e86vac lineage. The most abundant sequence was retained for analysis (green, e86vac), while less abundant sequences reflecting single base sequencing errors were excluded (light orange). Double base sequencing errors (dark orange) are also excluded, as they are in turn less abundant than single bases errors (and so on for triple base sequencing errors). A minimum of ten reads (dashed horizontal line) are required for a barcode to be included for further analysis.

Supplementary Fig. S2

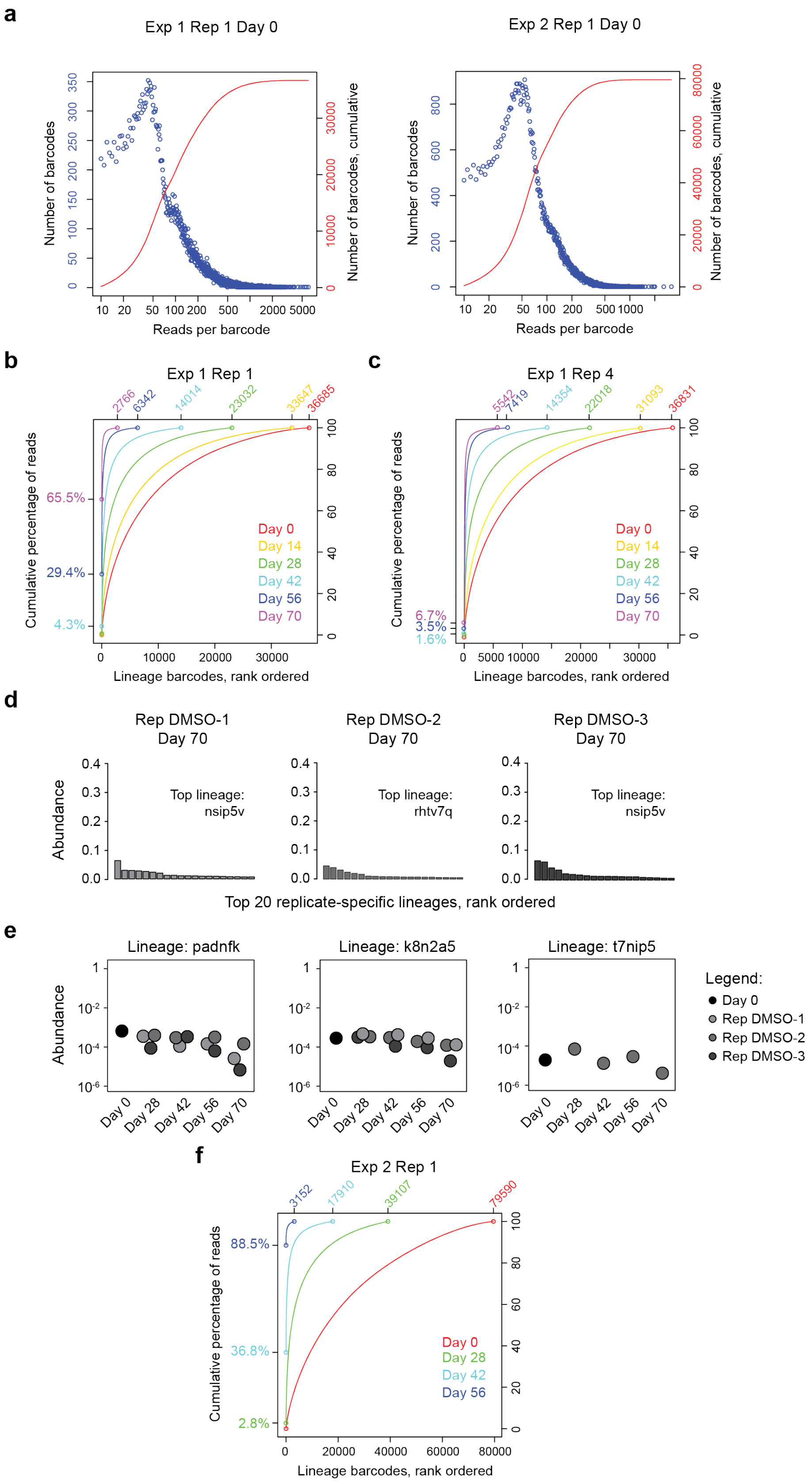

Figure S2.

a) Plots depict representative barcode distributions in pre-treatment (Day 0) cell populations for Experiments #1 and #2, depicted in similar manner as in Supplementary Fig. S1b. An average of 36,580 and 84,926 barcodes were detected in replicates from Experiments #1 and #2, respectively

b) Line plot compares barcode diversity across timepoints in a representative replicate containing a ‘jackpot’ lineage from Experiment #1. Barcodes were rank-ordered by frequency (x-axis) and the cumulative fraction of reads is shown on the y-axis. Abundance of the top ranking barcode for a given day in the indicated replicates is noted on the left-hand axis, and the total number of lineage barcodes for a given day is noted above the plot.

c) Line plot as in panel (b) compares barcode diversity across timepoints in the non-‘jackpot’ Replicate #4 from Experiment #1.

d) Barplots depict relative abundances of the top 20 lineages in each Experiment #1 DMSO control replicate after 70 days of dasatinib treatment, per gDNA sequencing. The barcode ID of the dominant lineage in each replicate is indicated.

e) Dotplots depict relative abundances of indicated barcode lineages at successive timepoints in the DMSO control Experiment #1 replicates. Each plot shows data for a different lineage barcode that corresponds to the top lineage identified at day 70 in one of the dasatinib-treated replicates (see Figure 1d). The data show that the dasatinib-associated jackpot lineages did not display fitness in control/DMSO conditions, and decreased in abundance over the course of the experiment.

f) Line plot as in panel b compares barcode diversity across timepoints in (a) representative replicate containing a ‘jackpot’ lineage from Experiment #2.

Supplementary Fig. S3

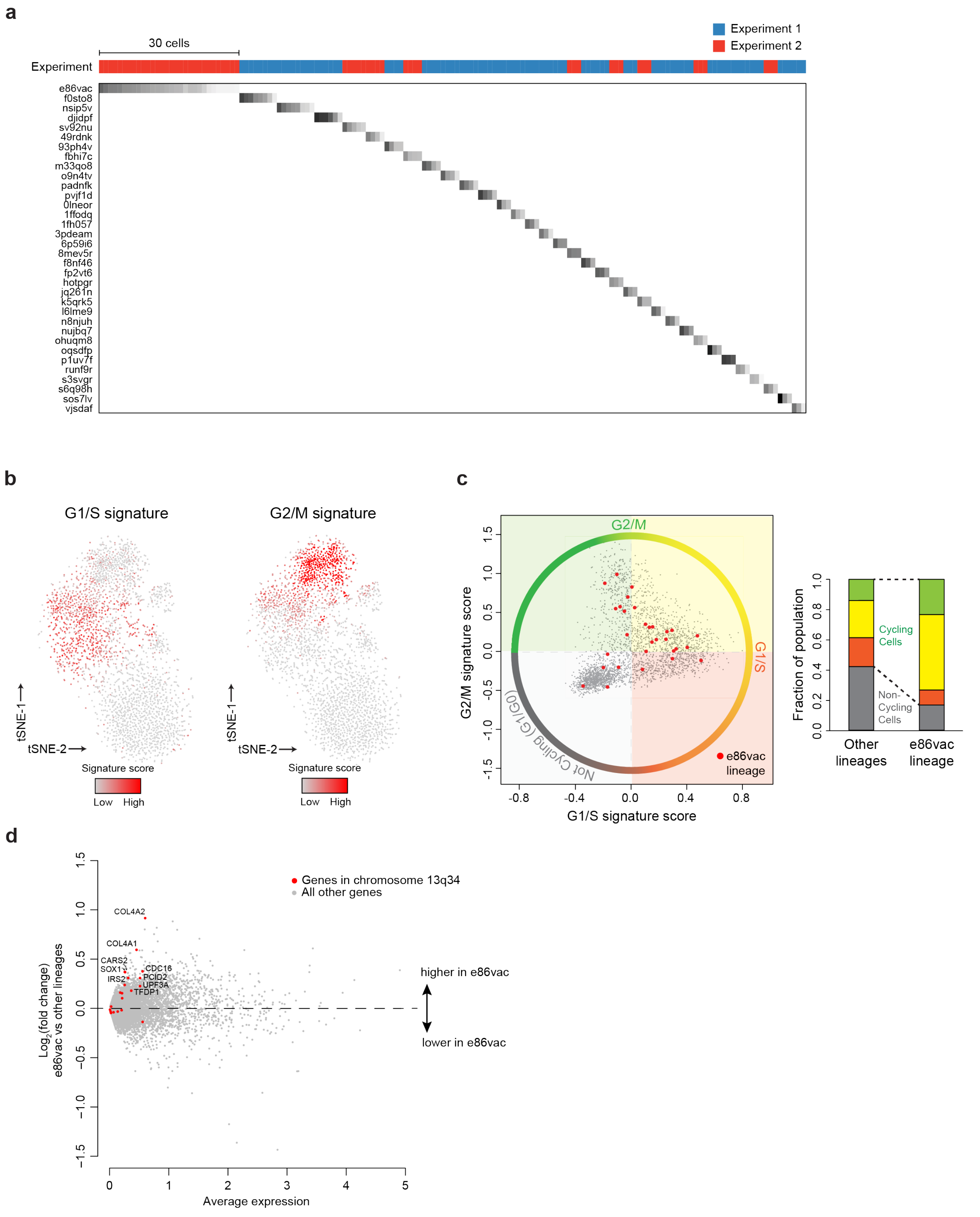

Figure S3.

a) Heatmap illustrates matching of single cells (x-axis) with lineage barcodes (y-axis). Only lineages which were identified in three or more cells are shown. Shades of gray indicate the number of supporting sequencing reads.

b) t-SNE plot displays single cells as in Figure 2b, colored according to their expression score of the indicated cell cycle gene signatures.

c) Scatter plot of single cells by their G1/S (x-axis) and G2/M (y-axis) expression scores. Red dots indicate those cells matching to the e86vac 'jackpot' lineage from Experiment #2. Stacked barplot quantifies the fraction of cells scoring for each cell cycle state for the e86vac lineage compared to all other lineages.

d) Scatter plot displays relative gene expression data levels of e86vac lineage cells relative to other lineages. Points represent individual genes (red = genes from chr13q34, gray = other genes) and are plotted by log_2-fold change (y-axis) versus average expression (x-axis).

Supplementary Figure 4

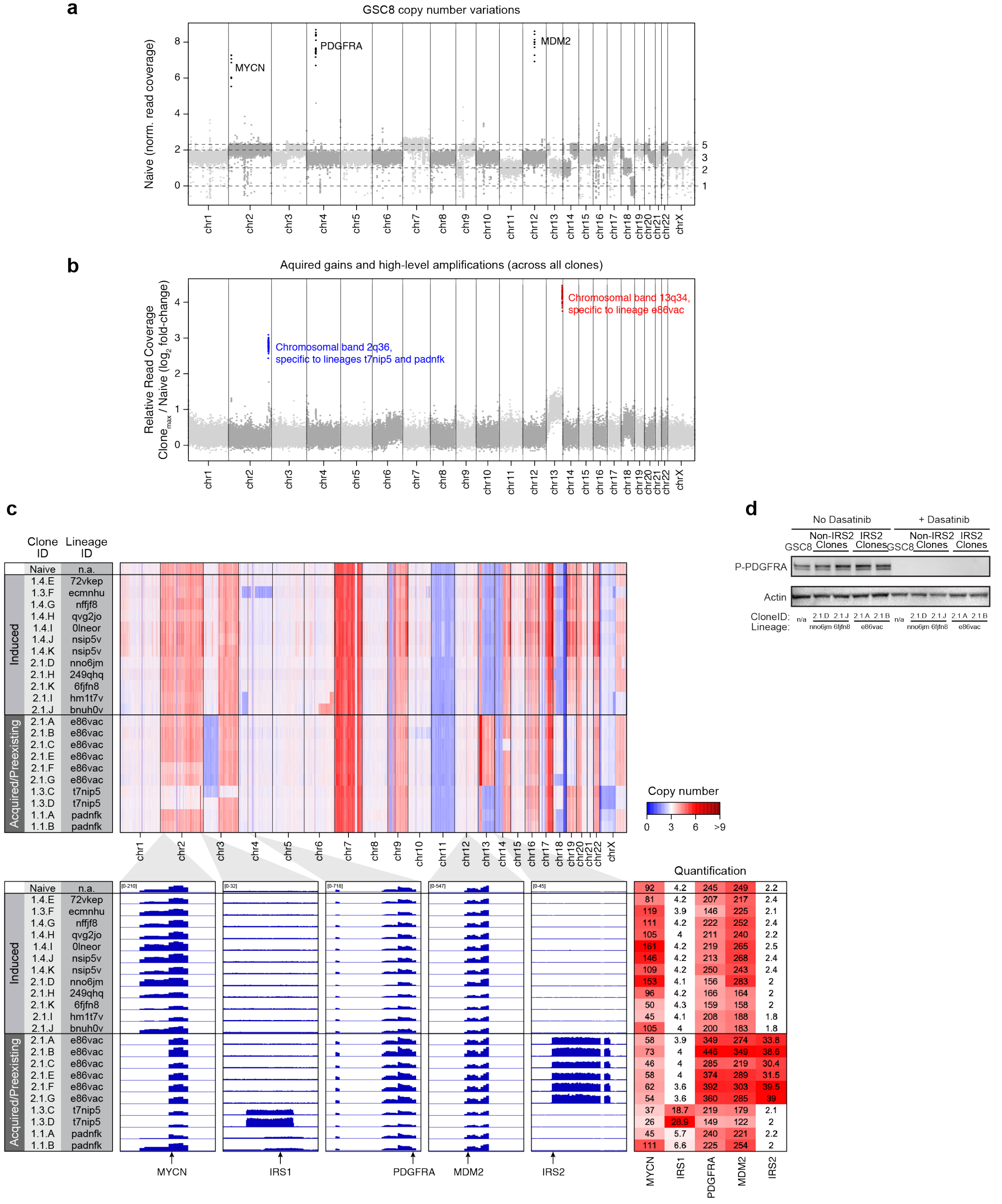

Clone ID	MYCN	IRS1	PDGFRA	MDM2	IRS2
Naive	92	4.2	245	249	2.2
1.4.E	81	4.2	207	217	2.4
1.3.F	119	3.9	146	225	2.1
1.4.G	111	4.2	222	252	2.4
1.4.H	105	4	211	240	2.2
1.4.I	161	4.2	219	265	2.5
1.4.J	146	4.2	213	268	2.4
1.4.K	109	4.2	250	243	2.4
2.1.D	153	4.1	156	283	2
2.1.H	96	4.2	166	164	2
2.1.K	50	4.3	159	158	2
2.1.I	45	4.1	208	188	1.8
2.1.J	105	4	200	183	1.8
2.1.A	58	3.9	349	274	33.8
2.1.B	73	4	446	349	38.6
2.1.C	46	4	285	219	30.4
2.1.E	58	4	374	289	31.5
2.1.F	62	3.6	392	303	39.5
2.1.G	54	3.6	360	285	39
1.3.C	37	18.7	219	179	2.1
1.3.D	26	28.9	149	122	2
1.1.A	45	5.7	240	221	2.2
1.1.B	111	6.6	225	254	2

Figure S4.

a) Genome plot of low-pass whole genome sequencing (WGS) read coverage within 50kb windows reveals that parental, dasatinib-naïve GSC8s display high-level amplifications at the PDGFRA, MYCN and MDM2 loci as well as a variety of other chromosomal or sub-chromosomal copy number alterations.

b) Genome plot depicts low-pass WGS read coverage of the dasatinib-resistant clonal cultures relative to parental cells shown in panel a. For each 50kb window only the maximum value of the 21 clonally-derived cultures is plotted. This analysis revealed that the Chr2q36 and Chr13q34 loci amplifications seen in lineages t7nip5/padnfk and e86vac, respectively, were the only prominent high level amplifications across all clonal cultures.

c) Heatmap depicting CNVs across dasatinib-naïve parental cells and all profiled clonal cultures revealed isolated chromosomal gains and losses, but did not identify any other recurrent copy number alterations within the dasatinib-exposed cultures. All clones retained the PDGFRA, MYCN, and MDM2 amplifications seen in the parental cells.

d) Western blot shows levels of phospho-PDGFRA and actin loading control. Input samples are as in Figure 5c and correspond to parental GSC8, non-jackpot clones from Experiment #2 and jackpot clones with IRS2 amplification grown in the presence of dasatinib.

Supplementary Fig. S5

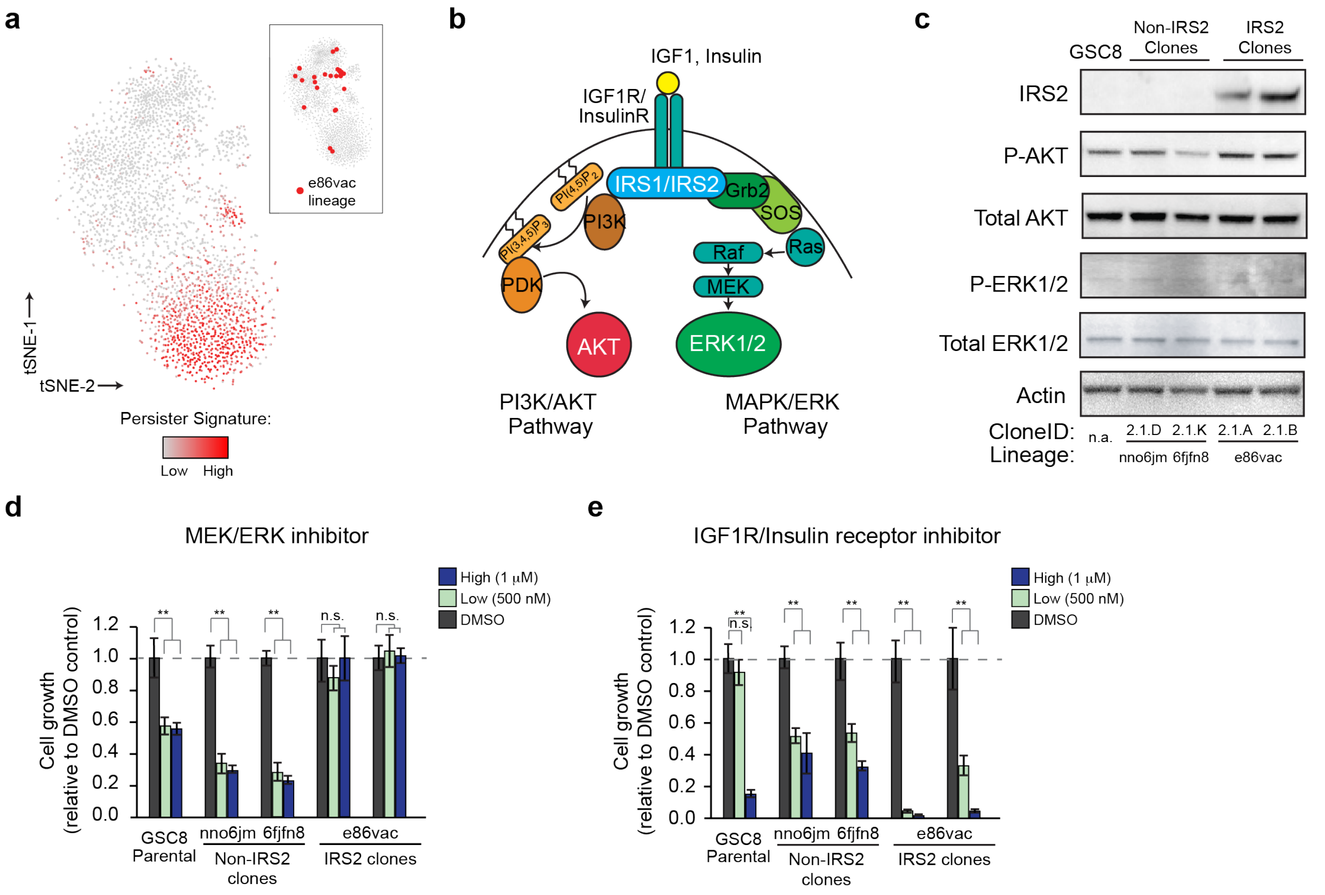

Figure S5.

a) t-SNE plot displays single cells from day 28 cultures as in Figure 2b, colored by their expression score for a gene signature derived from epigenetic persister cells[5]. Inset shows the same plot with cells from the e86vac jackpot lineage highlighted in red.

b) Schematic displays canonical downstream signaling pathways facilitated by IRS1/2.

c) Western blot shows levels of AKT, its active phospho-serine 463 isoform (P-AKT), ERK1/2, their active phospho-(Thr202/Tyr204) isoforms (P-ERK) and actin loading control. Input samples are as in Figure 5c and correspond to parental GSC8, non-jackpot clones from Experiment #2 and jackpot clones with IRS2 amplification grown in the presence of dasatinib. Positive control for ERK phosphorylation (not shown) was run alongside samples to verify phospho-antibody.

d) Barplots compare growth of parental GSC8, non-jackpot clones from Experiment #2 and jackpot clones from Experiment #2 with IRS2 amplification after at least 30 days of culture in

the presence of dasatinib. Dasatinib-containing cultures were treated with the indicated concentrations of MEK/ERK inhibitor or DMSO control (**, p<0.01 by two tailed student's t-test). The IRS2-amplified lineage is not dependent on MEK/ERK signaling.

e) Barplots with standard error bars compare growth of cell populations as in panel d. Dasatinib-containing cultures were treated with the indicated concentrations of IGFR1/Insulin Receptor inhibitor or DMSO control (**, p<0.01 by two-tailed student's t-test). The IRS2-amplified lineage retains dependence on Insulin/IGFR signaling.

Supplementary Figure 6

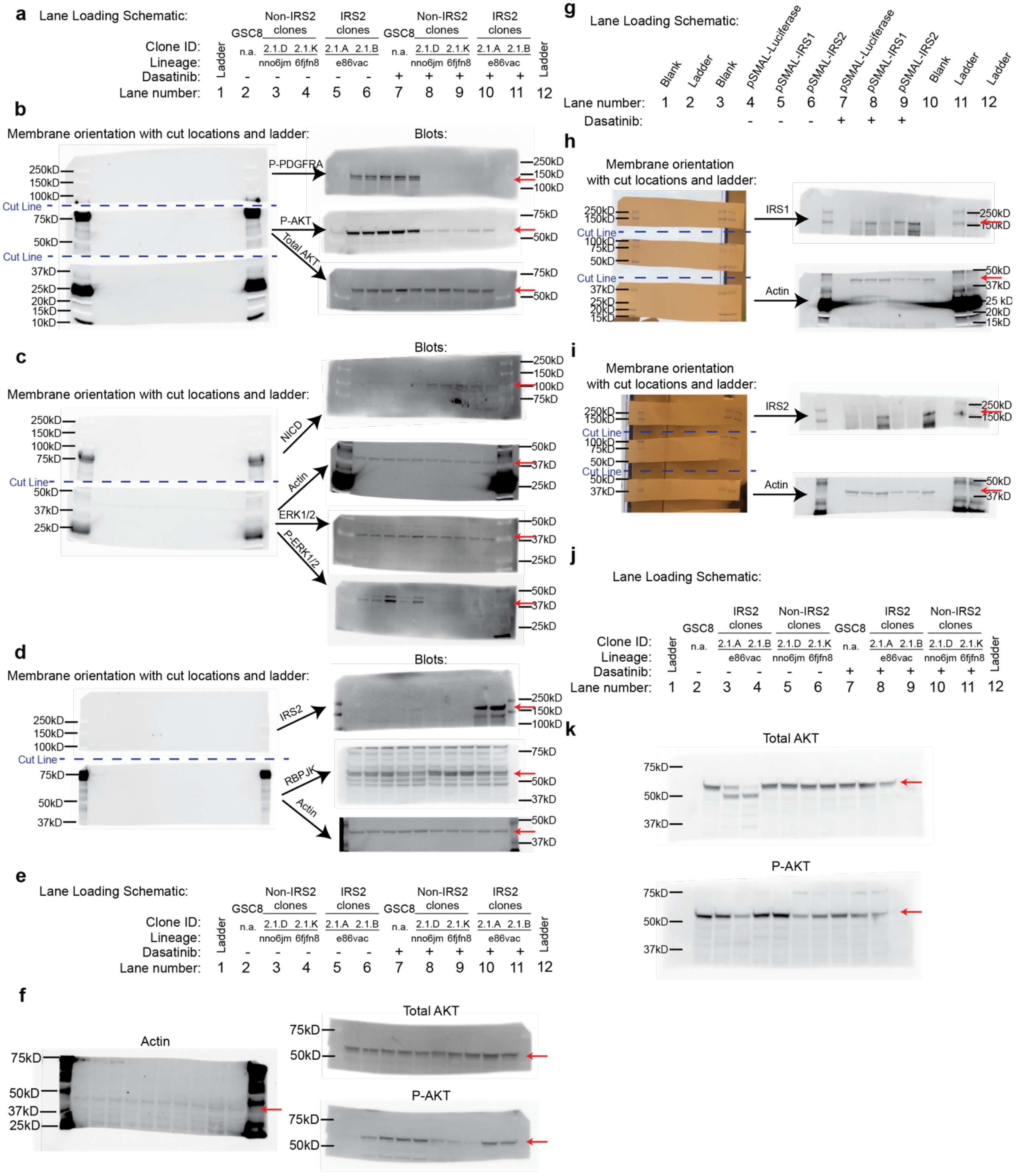

Figure S6.

a) Schematic depicts sample loading arrangement for panels b, c, and d. The prepared lysate was distributed between the three gels in identical amounts such that loading was identical between the three gels.

b-d) Uncropped Western blot data and depiction of gel layout including membrane cut sites and ladder labels for gel replicate 1 (b), 2 (c) and 3 (d). Data from these blots are contained in the following display items: Fig. 5c, Fig. S4d, Fig S5c. Additionally some data from these blots were included in the densitometric analysis depicted in Fig 5h.

e) Schematic depicts sample loading arrangement for panel f.

f) Uncropped Western blot data including ladder labels for Fig. 5g, and some data from these blots were included in the densitometric analysis depicted in Fig 5h.

g) Schematic depicts sample loading arrangement for panel h and i. The prepared lysate was distributed between the two gels in identical amounts such that loading was identical between the two gels.

h-i) Uncropped Western blot data including membrane cut sites and ladder labels for gel replicate 1 (h) and 2 (i). Data from these blots are contained in Fig. 3d.

j) Schematic depicts sample loading arrangement for panel k. Note the altered sample arrangement of samples relative to S6a and S6e.

k) Uncropped Western blot data including ladder labels for data included in the densitometric analysis depicted in Fig 5h.
